# Supplementary material for: Induction of Rosette-to-Lumen stage embryoids using reprogramming paradigms in ESCs
Source: Nat Commun. 2021 Dec 16;12:7322. doi: 10.1038/s41467-021-27586-w (PMC8677818; doi:10.1038/s41467-021-27586-w)
Supplement: Supplementary file 1 — Supplementary Information [file 41467_2021_27586_MOESM1_ESM.pdf]

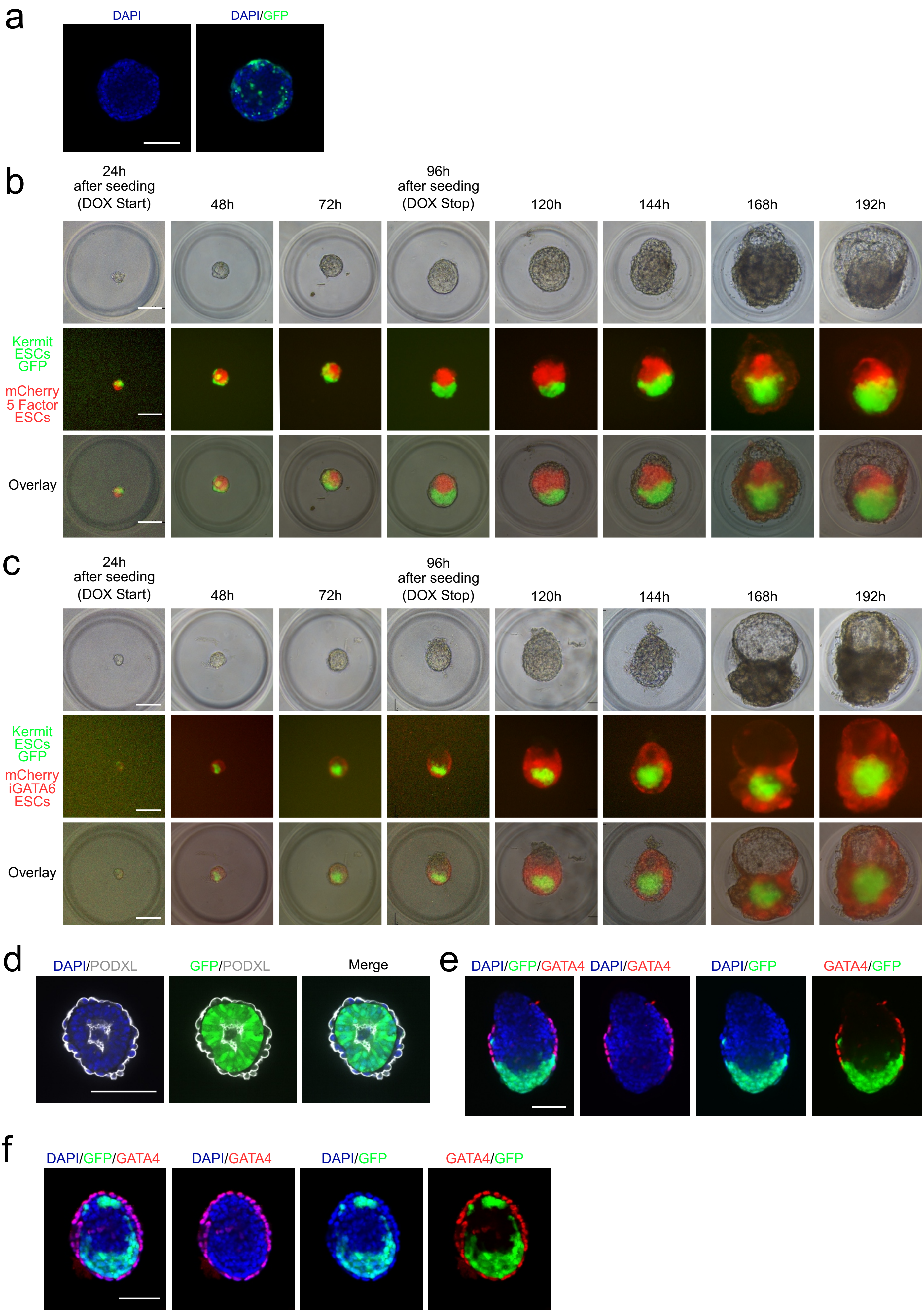
a
DAPI
DAPI/GFP
b
24h after seeding (DOX Start)
48h
72h
96h after seeding (DOX Stop)
120h
144h
168h
192h
Kermit ESCs GFP
mCherry 5 Factor ESCs
Overlay
c
24h after seeding (DOX Start)
48h
72h
96h after seeding (DOX Stop)
120h
144h
168h
192h
Kermit ESCs GFP
mCherry iGATA6 ESCs
Overlay
d
DAPI/PODXL
GFP/PODXL
Merge
e
DAPI/GFP/GATA4
DAPI/GATA4
DAPI/GFP
GATA4/GFP
f
DAPI/GFP/GATA4
DAPI/GATA4
DAPI/GFP
GATA4/GFP

Supplementary Figure 1 – Development of RtL-embryoids over time

a Image of aggregate composed of Kermit ESCs, 5 Factor ESCs and iGATA6 ESCs and cultured without DOX supplementation, showing random distribution and localization of GFP+ Kermit ESC and a complete lack of self-organization. GFP, green; DAPI, blue. Scale bar = 100 µm **b** Development of embryo-like structures built from Kermit ESCs, iGATA6 ESCs, and a 5 Factor ESC line transduced with a constitutive mCherry expression cassette. Detection of GFP originating from Kermit ESCs and mCherry from 5 Factor ESCs allows for visualization of self-organization into an epiblast-like (GFP+, green) and extraembryonic ectoderm-like (mCherry+, red) compartment. **c** Development of embryo-like structures build from Kermit ESCs, 5 Factor ESCs and an iGATA6 ESC line transduced with a constitutive mCherry cassette. Detection of GFP originating from Kermit ESCs and mCherry from iGATA6 ESCs allows for visualization of self-organization into an epiblast-like (GFP+, green) and visceral endoderm-like (mCherry+, red) compartment. Scale bars = 100 µm. **d** Aggregate composed of Kermit ESCs and iGATA6 ESCs readily display lumen formation, as indicated by staining against PODXL. Scale bar = 100 µm. GFP, green; PODXL, gray; DAPI, blue. **e** Examples of incomplete or **f** incorrectly assembled structures, that were excluded from analysis. Scale bars = 100 µm. GFP, green; GATA4, red; DAPI, blue. Experiments were repeated independently at least three times with similar results (**a, d, e, f**).

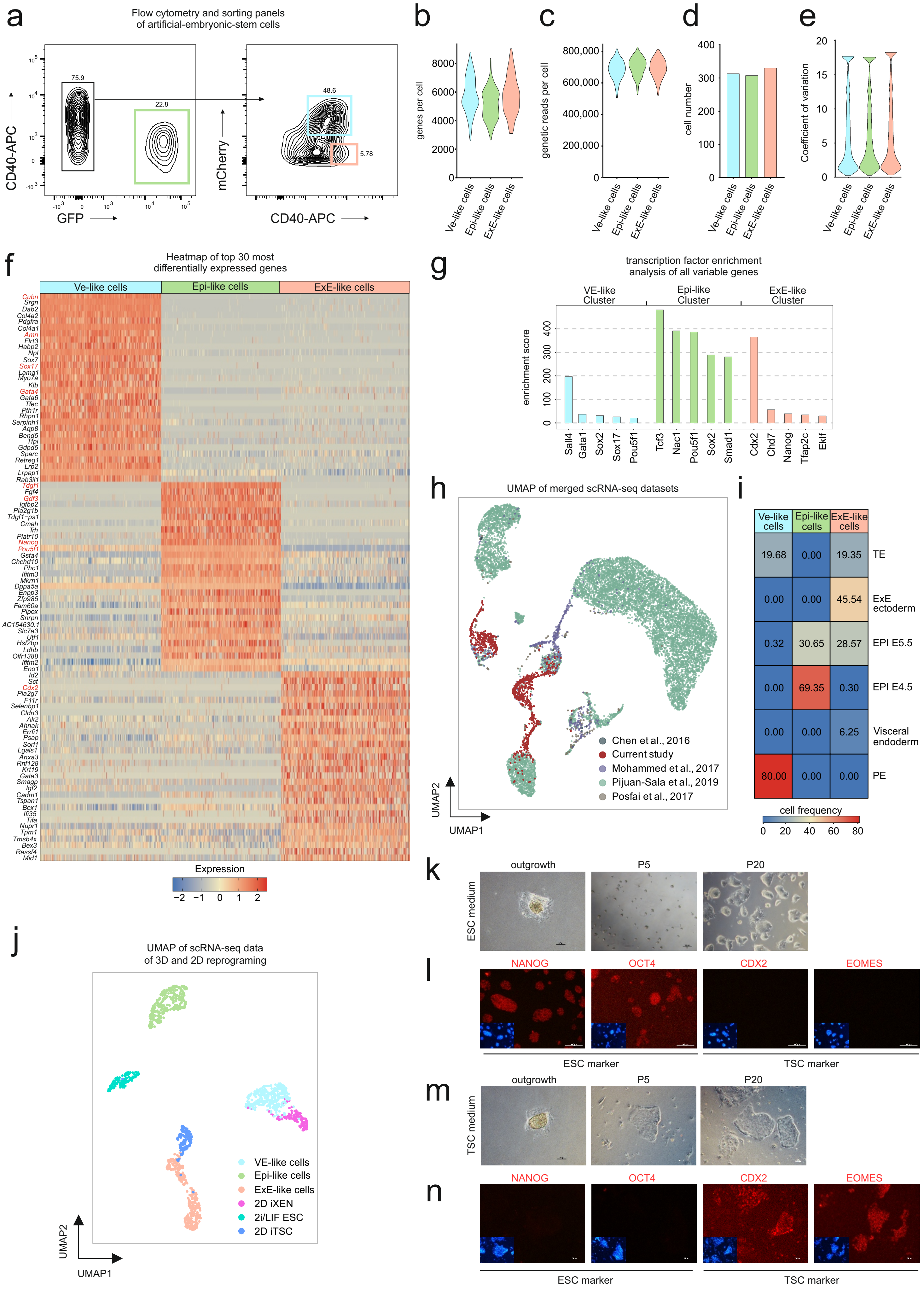

a
Flow cytometry and sorting panels of artificial-embryonic-stem cells
75.9
22.8
48.6
5.78
CD40-APC
GFP
mCherry
CD40-APC
b
genes per cell
c
genetic reads per cell
d
cell number
e
Coefficient of variation
Ve-like cells
Epi-like cells
ExE-like cells
f
Heatmap of top 30 most differentially expressed genes
Ve-like cells
Epi-like cells
ExE-like cells
Expression
g
transcription factor enrichment analysis of all variable genes
VE-like Cluster
Epi-like Cluster
ExE-like Cluster
enrichment score
Sall4
Gata1
Sox2
Sox17
Pou5f1
Tcf3
Nac1
Pou5f1
Sox2
Smad1
Cdx2
Chd7
Nanog
Tfap2c
Eklf
h
UMAP of merged scRNA-seq datasets
Chen et al., 2016
Current study
Mohammed et al., 2017
Pijuan-Sala et al., 2019
Posfai et al., 2017
UMAP1
UMAP2
i
Ve-like cells
Epi-like cells
ExE-like cells
19.68
0.00
19.35
TE
0.00
0.00
45.54
ExE ectoderm
0.32
30.65
28.57
EPI E5.5
0.00
69.35
0.30
EPI E4.5
0.00
0.00
6.25
Visceral endoderm
80.00
0.00
0.00
PE
cell frequency
j
UMAP of scRNA-seq data of 3D and 2D reprograming
VE-like cells
Epi-like cells
ExE-like cells
2D iXEN
2i/LIF ESC
2D iTSC
UMAP1
UMAP2
k
ESC medium
outgrowth
P5
P20
l
NANOG
OCT4
CDX2
EOMES
ESC marker
TSC marker
m
TSC medium
outgrowth
P5
P20
n
NANOG
OCT4
CDX2
EOMES
ESC marker
TSC marker

Supplementary Figure 2 – Flow cytometry, scRNA-Seq and stem cell derivation from RtL-embryoids

a Flow cytometry and sorting panels of cells from RtL-embryoids (light red sorting gate ExE-like cells/CD40-APC+; light green sorting gate Epi-like cells/ GFP+; light blue sorting gate VE-like cell/mCherry+). **b – e** scRNA-seq library statistic of data obtained from SMART-seq2 technology. **f** Heatmap showing expression of top 50 differentially expressed genes of VE-like cluster, Epi-like cluster, and ExE-like cluster. Genes are represented in rows, and cells in columns. **g** Transcription factor enrichment analysis showing enrichment score of key transcription factors for VE-like, Epi-like, and ExE-like clusters. **h** UMAP from Fig. 2f identifying original datasets[25,26,27,28] for each cell. **i** Heatmap of cell percentages from RtL-embryoid clusters mapped to the respective cell populations in the reference dataset of Fig. 2f. **j** UMAP of scRNA-seq data of 3D and 2D reprograming **k – n** Derivation and characterization of ES- and TS- cells obtained by outgrowth culture from RtL-embryoids. NANOG/OCT4/CDX2/EOMES, red; Nuclei staining: Hoechst33342, blue. Scale bars = 100 µm. Source data of **f**, **g** and **j** are provided as a Source Data file. Experiments were repeated independently at least three times with similar results (**k - n**).

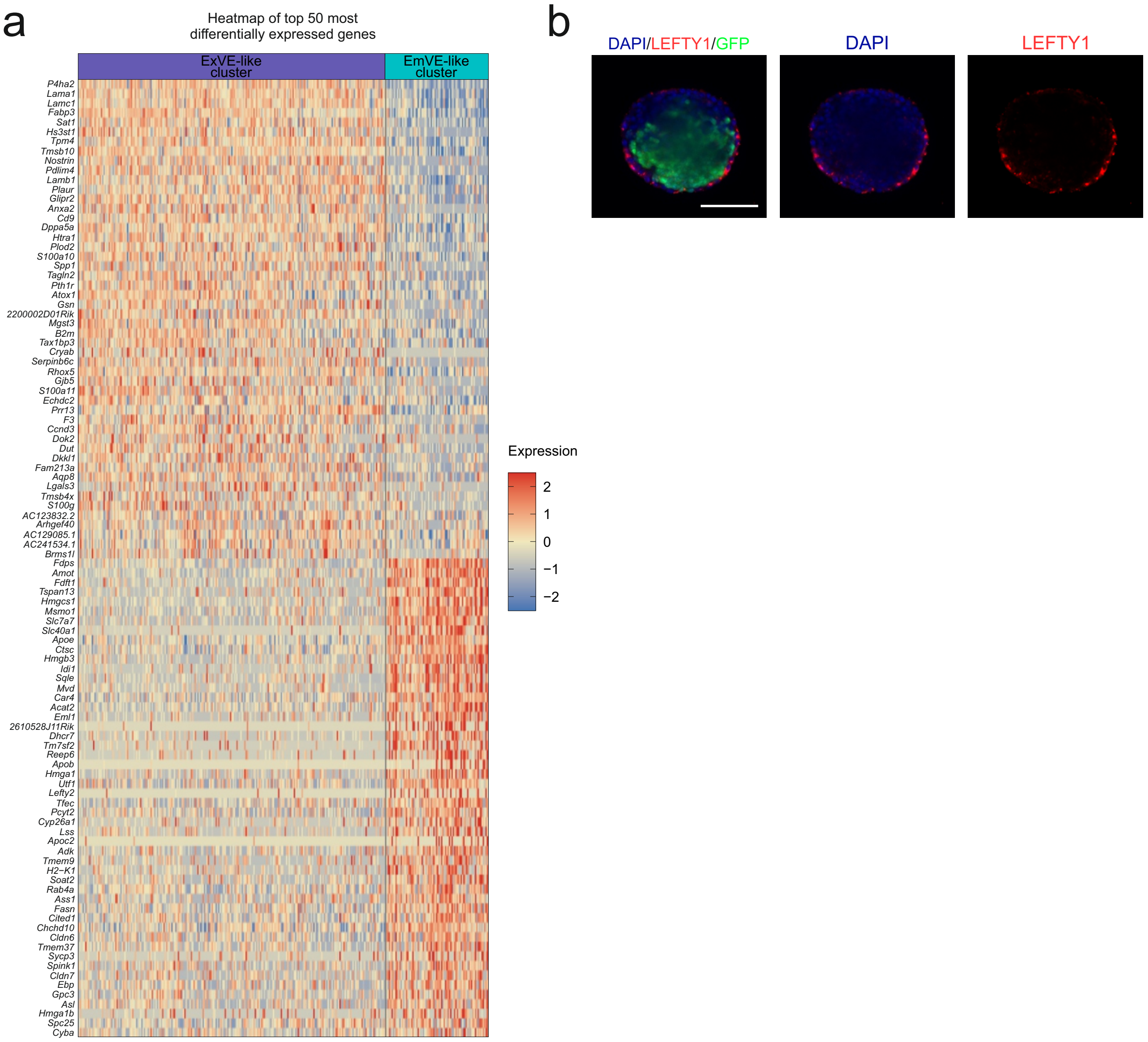
a
Heatmap of top 50 most differentially expressed genes
ExVE-like cluster
EmVE-like cluster
P4ha2
Lama1
Lamc1
Fabp3
Sat1
Hs3st1
Tpm4
Tmsb10
Nostrin
Pdlim4
Lamb1
Plaur
Glipr2
Anxa2
Cd9
Dppa5a
Htra1
Plod2
S100a10
Spp1
Tagln2
Pth1r
Atox1
Gsn
2200002D01Rik
Mgst3
B2m
Tax1bp3
Cryab
Serpinb6c
Rhox5
Gjb5
S100a11
Echdc2
Prr13
F3
Ccnd3
Dok2
Dut
Dkkl1
Fam213a
Aqp8
Lgals3
Tmsb4x
S100g
AC123832.2
Arhgef40
AC129085.1
AC241534.1
Brms1l
Fdps
Amot
Fdft1
Tspan13
Hmgcs1
Msmo1
Slc7a7
Slc40a1
Apoe
Ctsc
Hmgb3
Idi1
Sqle
Mvd
Car4
Acat2
Eml1
2610528J11Rik
Dhcr7
Tm7sf2
Reep6
Apob
Hmga1
Utf1
Lefty2
Tfec
Pcyt2
Cyp26a1
Lss
Apoc2
Adk
Tmem9
H2−K1
Soat2
Rab4a
Ass1
Fasn
Cited1
Chchd10
Cldn6
Tmem37
Sycp3
Spink1
Cldn7
Ebp
Gpc3
Asl
Hmga1b
Spc25
Cyba
Expression
2
1
0
−1
−2
b
DAPI/LEFTY1/GFP
DAPI
LEFTY1

Supplementary Figure 3 - Heatmap of variable genes of VE-like subclusters

a Heatmap displaying the top 50 differentially expressed genes for cells of the ExVE-like cluster and EmVE-like cluster. **b** LEFTY1 expression throughout, but predominantly restricted to the EmVE in aggregates with a weak contribution of ExE-like tissue. GFP, green; LEFTY1, red; DAPI, blue. Scale bars = 100 µm. Source data of **a** are provided as a Source Data file. Experiments were repeated independently at least three times with similar results (**b**).

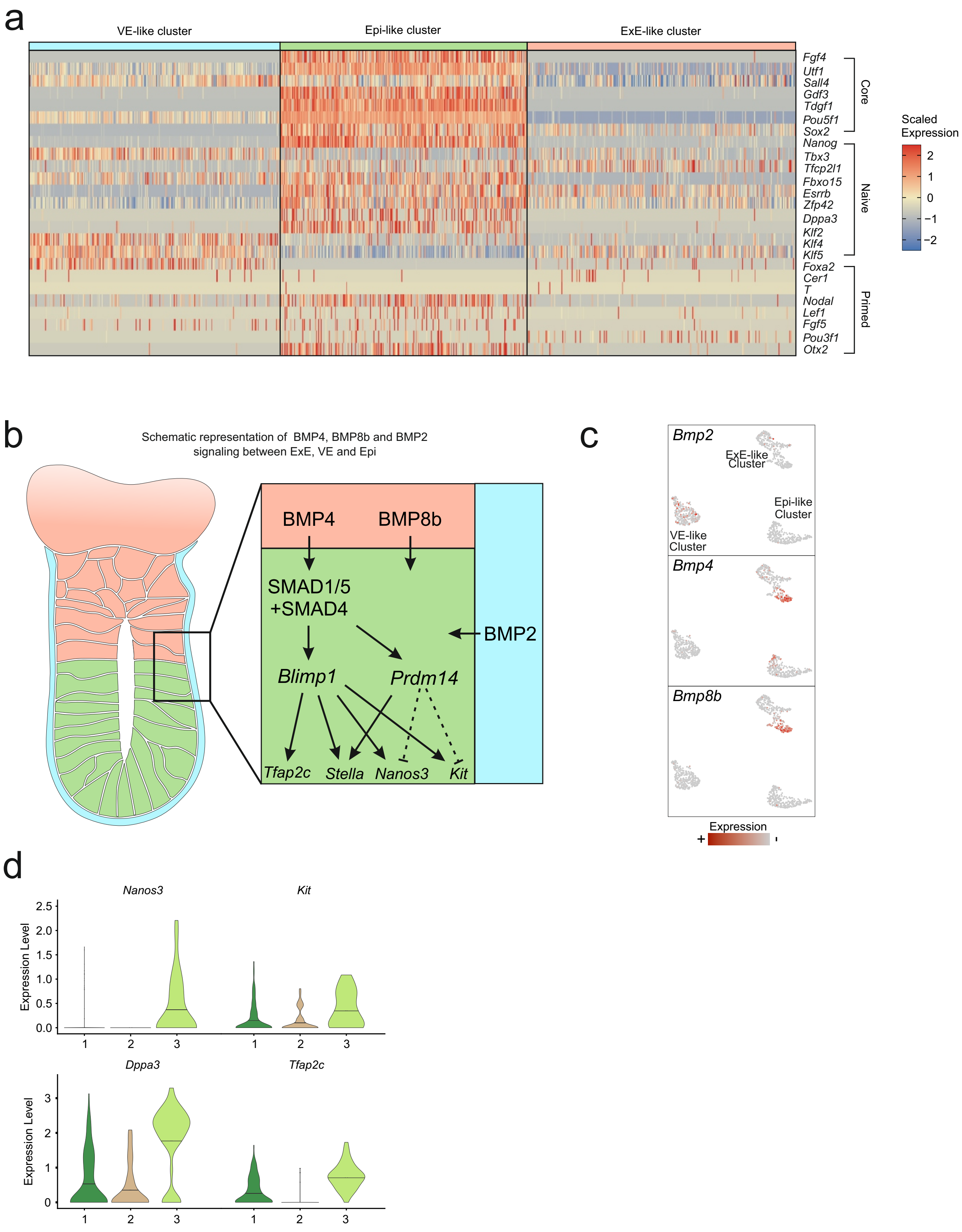
a
VE-like cluster
Epi-like cluster
ExE-like cluster
Fgf4
Utf1
Sall4
Gdf3
Tdgf1
Pou5f1
Sox2
Nanog
Tbx3
Tfcp2l1
Fbxo15
Esrrb
Zfp42
Dppa3
Klf2
Klf4
Klf5
Foxa2
Cer1
T
Nodal
Lef1
Fgf5
Pou3f1
Otx2
Core
Naive
Primed
Scaled Expression
2
1
0
−1
−2
b
Schematic representation of BMP4, BMP8b and BMP2 signaling between ExE, VE and Epi
BMP4
BMP8b
SMAD1/5 +SMAD4
BMP2
Blimp1
Prdm14
Tfap2c
Stella
Nanos3
Kit
c
Bmp2
ExE-like Cluster
Epi-like Cluster
VE-like Cluster
Bmp4
Bmp8b
Expression
+
-
d
Nanos3
Kit
Dppa3
Tfap2c
Expression Level
2.5
2.0
1.5
1.0
0.5
0.0
3
2
1
0
1
2
3

Supplementary Figure 4 – RtL-embryoids show indications for the induction of the molecular signaling cascade responsible for PGC specification.

a Heatmap showing expression of core-, naïve-, and primed-pluripotency factors across cells of all three compartments comprising RtL-embryoids. **b** Schematic representation of signaling events leading to PGC specification in epiblast of murine embryos starting between E4.5 – E5.5 with the secretion of BMP4, BMP8b from the ExE and BMP2 from the VE. **c** Featureplots mapping expression of Bmp2 in cells of the VE-like cluster and Bmp4 and Bmp8b in cells of ExE-like cluster. **d** Violinplots showing distinct subpopulation of cells expressing marker genes of PGC specification *Nanos3*, *Kit*, *Dppa3* (*Stella*), and *Tfap2c*, identifying Epi-like subcluster 3 as cells displaying PGC-like cell fate. Source data of **d** are provided as a Source Data file.

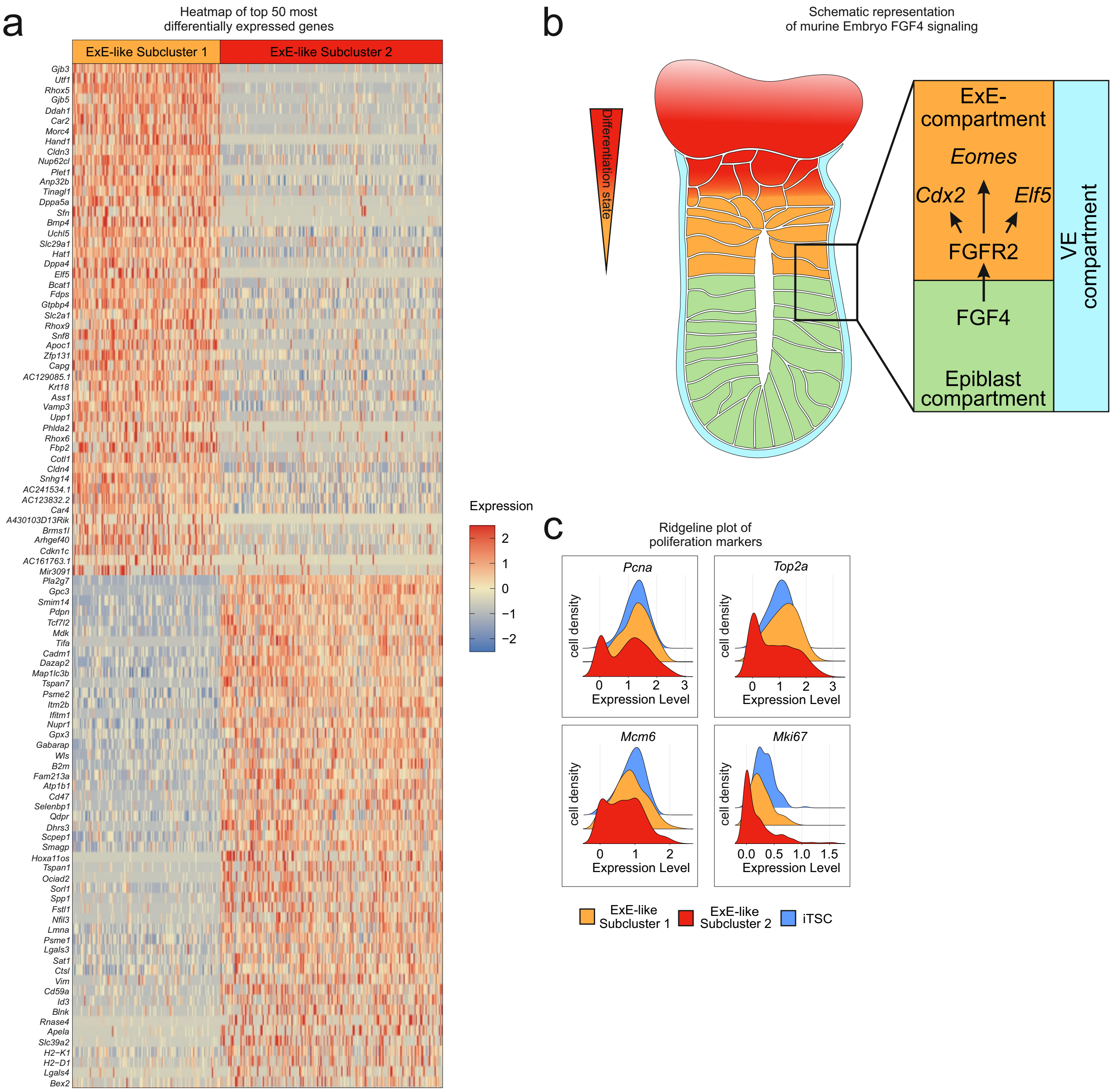
a
Heatmap of top 50 most differentially expressed genes
ExE-like Subcluster 1
ExE-like Subcluster 2
Expression
b
Schematic representation of murine Embryo FGF4 signaling
Differentiation state
ExE-compartment
Eomes
Cdx2
Elf5
FGFR2
FGF4
Epiblast compartment
VE compartment
c
Ridgeline plot of poliferation markers
Pcna
Top2a
Mcm6
Mki67
cell density
Expression Level
ExE-like Subcluster 1
ExE-like Subcluster 2
iTSC

Supplementary Figure 5 – Detailed analysis and characterization of ExE-like subcluster 1 and 2

a Heatmap depicting the top 50 differentially expressed genes of the two ExE-Subclusters. **b** Schematic representation of FGF4 signaling and downstream targets in ExE compartment in murine embryos ~E5.5. **c** Ridgeline plots of proliferation marker gene expression in the two ExE-like Subcluster and iTSCs obtained from FGF4/Heparin supplemented reprogramming in 2D mono-culture, highlighting the stem cell character of ExE-like Subcluster 1. Source data of **a** are provided as a Source Data file.
