# Supplementary material for: Induction of Rosette-to-Lumen stage embryoids using reprogramming paradigms in ESCs
Source: Nat Commun. 2021 Dec 16;12:7322. doi: 10.1038/s41467-021-27586-w (PMC8677818; doi:10.1038/s41467-021-27586-w)
Supplement: Supplementary file 3 — Reporting Summary [file 41467_2021_27586_MOESM3_ESM.pdf]

nature research

Corresponding author(s): Prof. Dr. Hubert Schorle

Last updated by author(s): Nov 10, 2021

Reporting Summary

Nature Research wishes to improve the reproducibility of the work that we publish. This form provides structure for consistency and transparency in reporting. For further information on Nature Research policies, see our Editorial Policies and the Editorial Policy Checklist.

Statistics

For all statistical analyses, confirm that the following items are present in the figure legend, table legend, main text, or Methods section.

n/a	Confirmed	
☐	☒	The exact sample size (n) for each experimental group/condition, given as a discrete number and unit of measurement
☐	☒	A statement on whether measurements were taken from distinct samples or whether the same sample was measured repeatedly
☐	☒	The statistical test(s) used AND whether they are one- or two-sided<br>*Only common tests should be described solely by name; describe more complex techniques in the Methods section.*
☒	☐	A description of all covariates tested
☐	☒	A description of any assumptions or corrections, such as tests of normality and adjustment for multiple comparisons
☐	☒	A full description of the statistical parameters including central tendency (e.g. means) or other basic estimates (e.g. regression coefficient) AND variation (e.g. standard deviation) or associated estimates of uncertainty (e.g. confidence intervals)
☐	☒	For null hypothesis testing, the test statistic (e.g. F, t, r) with confidence intervals, effect sizes, degrees of freedom and P value noted<br>*Give P values as exact values whenever suitable.*
☒	☐	For Bayesian analysis, information on the choice of priors and Markov chain Monte Carlo settings
☐	☒	For hierarchical and complex designs, identification of the appropriate level for tests and full reporting of outcomes
☐	☒	Estimates of effect sizes (e.g. Cohen's d, Pearson's r), indicating how they were calculated

Our web collection on statistics for biologists contains articles on many of the points above.

Software and code

Policy information about availability of computer code

Data collection

Flow cytometry and sorting were performed using FACS DIVA (v 8.0.1 on BD Aria III BD Pharmingen). Sequencing data were collected on a NextSeq500 .
Seurat (R package) (version 3.1.2, 4.0.3, 4.0.1)
ClusterProfiler (R package) (version 3.14.3)
AUCell (R package) (version 1.10.1)
GraphPad Prism (software) (version 8.0.2)
NicheNet (R package) (version 1.0.0)
ggplot2 (R package) (version 3.3.2)
pheatmap (R package) (version 1.0.12)
tximport (R package) (version 1.16.1)
Kallisto (software) (version 0.44.0)
FACS DIVA (software) (version 8.0.1)
Fiji (software) (version 1.53c)
FlowJo (software) (version 10.6.1)
Powerpoint (software) (version 2110)

Data analysis

All the computational analyses were performed using R programming languages. Scripts of key steps can be found at [http://github.com/schultzelab/Rosette-to-Lumen-stage-embryoids]. Additionally, our code to reproduce the analysis can be accessed via FASTGenomics [https://beta.fastgenomics.org/analyses/detail-analysis-9301d957888c40538020f90428bcc763#Result&analysis].
RNA-Seq libraries were subjected to initial quality control using FASTQC [http://www.bioinformatics.babraham.ac.uk/projects/fastqc] (version 0.11.7) implemented in a scRNA pre-processing pipeline (docker image and scripts available at [https://hub.docker.com/r/pwlb/rna-seq-

pipeline-base/] (version 0.1.1); [https://bitbucket.org/limes_bonn/bulk-rna-kallisto-qc/src/master/] (version 0.2.1))

For manuscripts utilizing custom algorithms or software that are central to the research but not yet described in published literature, software must be made available to editors and reviewers. We strongly encourage code deposition in a community repository (e.g. GitHub). See the Nature Research guidelines for submitting code & software for further information.

Data

Policy information about availability of data

All manuscripts must include a data availability statement. This statement should provide the following information, where applicable:

- Accession codes, unique identifiers, or web links for publicly available datasets
- A list of figures that have associated raw data
- A description of any restrictions on data availability

Data Availability:
Raw sequencing data of mouse Smart-seq2 generated in this study have been deposited in the Gene Expression Omnibus (GEO) database under accession code GSE188394 [http://www.ncbi.nlm.nih.gov/geo/query/acc.cgi?acc=GSE188394]. Additionally, data are deposited via FASTGenomics [https://beta.fastgenomics.org/d/200474]. The FASTGenomics platform also provides Seurat objects of the datasets generated in this study.
Source data are provided with this paper. The publicly available datasets analyzed during the current study are available from the GEO- and ArrayExpress repository.
GSE84892 [https://www.ncbi.nlm.nih.gov/geo/query/acc.cgi?acc=GSE84892]25
GSE74155 [https://www.ncbi.nlm.nih.gov/geo/query/acc.cgi?acc=GSE74155]26
GSE100597 [https://www.ncbi.nlm.nih.gov/geo/query/acc.cgi?acc=GSE100597]27
E-MTAB-6967 [https://www.ebi.ac.uk/arrayexpress/experiments/E-MTAB-6967/]28
The mouse genome used for kallisto alignment is available from the GENCODE Project.
GRCm38, Gencode vM16 primary assembly [https://www.gencodegenes.org/mouse/release_M16]

Code Availability:
All the computational analyses were performed using R programming languages. Scripts of key steps can be found at [http://github.com/schultzelab/Rosette-to-Lumen-stage-embryoids]. Additionally, our code to reproduce the analysis can be accessed via FASTGenomics [https://beta.fastgenomics.org/analyses/detail-analysis-9301d957888c40538020f90428bcc763#Result&analysis].

Field-specific reporting

Please select the one below that is the best fit for your research. If you are not sure, read the appropriate sections before making your selection.

☒ Life sciences ☐ Behavioural & social sciences ☐ Ecological, evolutionary & environmental sciences

For a reference copy of the document with all sections, see nature.com/documents/nr-reporting-summary-flat.pdf

Life sciences study design

All studies must disclose on these points even when the disclosure is negative.

Sample size	RtL-embryoids were generated in 3D Petri Dishes in each well of a 12 well plate, resulting in >700 correctly assembled RtL-embryoids per 12 well plate (in twelve 3D Petri Dishes). In general, RtL-embryoids from at least 6 3D Petri Dishes were used per experimental replicate.
Data exclusions	Aggregates that displayed incomplete self-assembly into Epi-, VE- and ExE-like compartments were excluded from the experiments, as described in the manuscript. RtL-embryoids that were lost during medium exchange were also excluded. For data quality control in scRNA-seq we excluded the cells with less than 2500 expressed genes and less than 500,000 aligned sequencing reads.
Replication	All attempts at replicating the described methods and experimental outcomes were successful. RNA-Sequencing was performed only once, with >600 correctly assembled aggregates from 3 biological replicates (200 correctly assembled structures from each replicate).<br>All other experiments presented in this study were performed at least three times, yielding reproducible results.
Randomization	Correctly assembled RtL-embryoids showing complete self-organization into Epi-, VE- and ExE-like tissues were picked and used for analysis.
Blinding	The investigators were not blinded during experiments, as this study compares RtL-embryoid with natural embryos and the experiments were descriptive in nature.

Reporting for specific materials, systems and methods

We require information from authors about some types of materials, experimental systems and methods used in many studies. Here, indicate whether each material, system or method listed is relevant to your study. If you are not sure if a list item applies to your research, read the appropriate section before selecting a response.

Materials & experimental systems

n/a	Involved in the study
☐	☒ Antibodies
☐	☒ Eukaryotic cell lines
☒	☐ Palaeontology and archaeology
☒	☐ Animals and other organisms
☒	☐ Human research participants
☒	☐ Clinical data
☒	☐ Dual use research of concern

Methods

n/a	Involved in the study
☒	☐ ChIP-seq
☐	☒ Flow cytometry
☒	☐ MRI-based neuroimaging

Antibodies

Antibodies used

Goat polyclonal anti-CDX2 (Santa Cruz; sc-19478; Dilution: 1:200)
Goat polyclonal anti-GATA4 (Santa Cruz: sc-1237; Dilution: 1:400)
Goat-polyclonal anti-CD40 (R&D Systems: AF440; Dilution: 1:300)
Goat-polyclonal anti-LEFTY1 (R&D Systems: AF746; Dilution: 1:200)
Rabbit-polyclonal anti-p44/42 MAPK (Erk1/2) (Cell signaling: #9101; Dilution: 1:100)
Mouse-polyclonal anti-OCT6 (Absea: 060204E04; Dilution: 1:10)
Mouse-polyclonal anti-ESRRB (Perseus Proteomics :PP-H6705-00; Dilution: 1:200)
Rabbit-polyclonal anti-NANOG (ReproCell: RCAB002P-F; Dilution: 1:300)
Rabbit-polyclonal anti-EOMES (Abcam: ab23345; Dilution: 1:400)
Goat-polyclonal anti-KLF4 (R&D: AF3158; Dilution: 1:400)
Rat-polyclonal anti-PODXL (R&D: MAB1556; Dilution: 1:300)
Goat-polyclonal anti-OTX2 (R&D: AF1979; Dilution: 1:400)
Rabbit-polyclonal anti-GATA3 (Abcam: ab199428; Dilution: 1:300)
Mouse-polyclonal anti-OCT4 (Santa Cruz: sc-5279; Dilution: 1:300)
Alexa Fluor 488-Phalloidin (Invitrogen: A12379; Concentration: 5 units/ml)
Alexa Fluor 594-Phalloidin (Invitrogen: A12381; Concentration: 5 units/ml)
Donkey polyclonal secondary antibody to Goat IgG-H&L (Alexa Fluor 594) (Abcam: ab150132; Dilution: 1:500)
Chicken polyclonal secondary antibody to Goat IgG-H&L (Alexa Fluor 647) (Invitrogen: A-21469; Dilution: 1:500)
Goat polyclonal secondary antibody to Rabbit IgG-H&L (Alexa Fluor 594) (Invitrogen: A-11012; Dilution: 1:500)
Goat polyclonal secondary antibody to Rat IgG-H&L (Alexa Fluor 488) (Invitrogen: A-11006; Dilution: 1:500)
Goat polyclonal secondary antibody to Mouse IgG-H&L (Alexa Fluor 488) (Invitrogen: A-11001; Dilution: 1:500)
Donkey polyclonal secondary antibody to Mouse IgG-H&L (Alexa Fluor 594) (Invitrogen: A-21203; Dilution: 1:500)
Chicken polyclonal secondary antibody to Rat IgG-H&L (Alexa Fluor 647) (Invitrogen: A-21472; Dilution: 1:500)
Goat polyclonal secondary antibody to Rabbit IgG-H&L (Alexa Fluor 488) (Invitrogen: A-11008; Dilution: 1:500)

Validation

Goat polyclonal anti-CDX2 (Santa Cruz; sc-19478): IF staining: PMID: # 20525899 Januário, DA. et al. 2010. Toxicol. Sci.
Goat polyclonal anti-GATA4 (Santa Cruz: sc-1237) IF staining: PMID: # 26893347 Yu, W. et al. 2016. Development
Goat-polyclonal anti-PKCz (Santa Cruz: sc-216-G) IF staining: PMID: # 22354172 Sakamori, R. et al. 2012. J. Clin. Invest.
Goat-polyclonal anti-CD40 (R&D Systems: AF440) FACS: PMID: # 32056544 Kaiser, F. et al. 2020. Placenta
Goat-polyclonal anti-LEFTY1 (R&D Systems: AF746) IF staining was successfully tested on E4.5 mouse embryos (see manufacturer's website)
Rabbit-polyclonal anti-p44/42 MAPK (Erk1/2) (Cell signaling: #9101) IF staining: PMID: #12925581 Corson, L.B. et al., 2003 Dev.
Mouse-polyclonal anti-OCT6 (Absea: 060204E04) IF staining: PMID: #32367046 Neagu, A. et al., 2020 Nat Cell Biol.
Mouse-polyclonal anti-ESRRB (Perseus Proteomics :PP-H6705-00) IF staining: PMID: #32367046 Neagu, A. et al., 2020 Nat Cell Biol.
Mouse-polyclonal anti-OCT4 (Santa Cruz: sc-5279): IF stainings performed on mouse embryonic stem cells can be found on manufacturer's website. The antibody has been referenced in more than 2000 publications.
Rabbit-polyclonal anti-NANOG (ReproCell: RCAB002P-F) IF staining: PMID: #32367046 Neagu, A. et al., 2020 Nat Cell Biol.
Rabbit-polyclonal anti-EOMES (Abcam: ab23345) IF staining: PMID: # 32056544 Kaiser, F. et al. 2020. Placenta
Goat-polyclonal anti-KLF4 (R&D: AF3158) IF staining: PMID: #32367046 Neagu, A. et al., 2020 Nat Cell Biol.
Alexa Fluor 488-Phalloidin (Invitrogen: A12379) IF staining: See manufacturer's website for references
Alexa Fluor 594-Phalloidin (Invitrogen: A12381) IF staining: See manufacturer's website for references
Rat-polyclonal anti-PODXL (R&D: MAB1556) IF staining: PMID: #32367046 Neagu, A. et al., 2020 Nat Cell Biol.
Goat-polyclonal anti-OTX2 (R&D: AF1979) IF staining: PMID: #32367046 Neagu, A. et al., 2020 Nat Cell Biol.
Rabbit-polyclonal anti-GATA3 (Abcam: ab199428) IF staining: PMID: # 32056544 Kaiser, F. et al. 2020. Placenta

Eukaryotic cell lines

Policy information about cell lines

Cell line source(s)

- Kermit ESCs were generated by our group from blastocysts of Oct3/4_GFP transgenic mice PMID: # 10646797 Yoshimizu, T. et al. 1999. Dev Growth Differ.
- KNUT1 ESCs were generated by our group and previously used and described in PMID: # 17661398 Peitz, M. et al. 2007. Genesis

	- 5 Factor ESCs were generated by our group and previously used and described in PMID: # 32056544 Kaiser, F. et al. 2020. Placenta<br>- iGATA6 ESCs were created by our group using lentiviral transduction with pCW57.1_Gata6, a gift from Constance Ciaudo (Addgene #73537) as described in PMID: #29396181 Ngondo, R. P. et al. 2018. Stem cell reports<br>- HEK 293T cell line (ATCC Order number #CRL-3216) was received from Dr. Michael Peitz.<br>- 5 Factor_mCherry and iGATA6_mCherry ESC lines were generated by our group entiviral transduction of the cell lines mentioned above with a lentiviral vector constitutively expressing mCherry
Authentication	All cell lines were validated by IF, FACS and scRNA-Seq
Mycoplasma contamination	All cell lines were tested negative for mycoplasma
Commonly misidentified lines (See ICLAC register)	No misidentified cell lines were used according to version 10 of the register of misidentified cell lines.

Flow Cytometry

Plots

Confirm that:

- [x] The axis labels state the marker and fluorochrome used (e.g. CD4-FITC).
- [x] The axis scales are clearly visible. Include numbers along axes only for bottom left plot of group (a 'group' is an analysis of identical markers).
- [x] All plots are contour plots with outliers or pseudocolor plots.
- [x] A numerical value for number of cells or percentage (with statistics) is provided.

Methodology

Sample preparation	For analysis of the 3D co-cultured cells by FACS and subsequent scRNA-SEQ RtL-embryoids were build from Kermit ESCs, 5Factor ESCs and iGATA6_mCherry ESCs and more than 600 correctly compartmented structures were identified (mCherry+ VE-like structure surrounding two inner compartments, a GFP+ Epi-like compartment next to an unstained ExE-like compartment). Correctly compartmented structures were pooled and dissociated into a single-cell suspension by incubation for 15 minutes in StemPro Accutase Cell Dissociation Reagent. Cells were passed through a 40 µm cell strainer and stained against CD40, thereby allowing for fluorescence-activated cell sorting of GFP+ (Kermit ESCs), mCherry+ (iGATA6 ESC derivates) and CD40+ (Alexa-647+) (5 Factor ESC derivates). Live/dead staining was performed using the Fixable Near-IR Dead Cell Stain Kit (Invitrogen; L34975) according to manufacturer's protocol. For the analysis of 2D mono-culture induced cell fates, Kermit ESCs were cultured in 2i/LIF supplemented ESC culture medium, iGATA6- and 5Factor ESCs were cultured in standard XENc medium or standard TSC culture medium respectively. Following transgene induction by DOX supplementation for three days and one day without DOX addition, the cells were harvested for analysis. Dissociation, FACS and scRNA-SEQ of 2D mono-cultured cells were performed using the same labeling, sorting and sequencing procedures as described before.
Instrument	BD FACS ARIA III
Software	BD FACS DIVA (data collection), FlowJo (analysis)
Cell population abundance	All sorted cells were identified and checked via SMART-Seq2 protocol
Gating strategy	All sample were gated on size by FSC-A/SSC-A. Doublets were removed with both FSC-H/FSC-A and SSC-H/SSC-A. Live dead staining was performed to exclude dead cells. Gating strategy for sorting of CD40-APC+, mCherry+ and GFP+ cell is displayed in Supplemental Figure 3A.

- [x] Tick this box to confirm that a figure exemplifying the gating strategy is provided in the Supplementary Information.
